# Quality of Life After Heart Transplantation: Instruments, Domains, and Variations by Time, Age, and Gender

**DOI:** 10.7759/cureus.112384

**Published:** 2026-07-10

**Authors:** Kush Shah, Jayottri Acharjee, Oluwatoyin Emaiye, Sowmya Sreekanth, Mekdes T Ambaye, Oluwabunmi J Adedoye, Siddharth Shah, Rebecca J Auta, Tavsimran S Luthra

**Affiliations:** 1 Medicine, University of Lancashire, Preston, GBR; 2 Medicine, Marche Polytechnic University, Ancona, ITA; 3 Orthopedics, University Hospital Crosshouse, Kilmarnock, GBR; 4 Cardiology, Presbyterian Hospital, New York, USA; 5 Internal Medicine, Sante Medical College, Addis Ababa, ETH; 6 Internal Medicine, Mater Dei Hospital, Msida, MLT; 7 Cardiology, Om Sairam Cardiac and Multispeciality Hospital, Bardoli, IND; 8 Neuroscience, Mater Dei Hospital, Msida, MLT; 9 Internal Medicine, Montefiore Medical Center, Wakefield Campus, Bronx, USA

**Keywords:** advanced heart failure, heart transplantation, mechanical circulatory support, outcome research, quality of life

## Abstract

Quality of life (QoL) has become a central outcome in heart transplantation, complementing traditional metrics of graft survival and clinical morbidity. As advances in surgical technique and immunosuppression have extended posttransplant longevity, understanding how patients function and experience daily life has gained increasing clinical relevance. The objective of this review is to synthesize contemporary evidence on QoL across the physical, psychological, social, and treatment-related domains, with particular emphasis on how these dimensions evolve over early, intermediate, and long-term follow-up. We summarize established tools for assessing QoL, highlight key determinants in each domain, and describe how factors such as exercise capacity, emotional recovery, social reintegration, and immunosuppressive burden contribute to the patient experience over time. Special attention is given to the limited but growing data on long-term survivors, whose QoL trajectories reveal both sustained benefits and emerging challenges decades after transplantation. Together, this review provides a comprehensive overview of current knowledge and outlines what clinicians and researchers can expect when evaluating QoL in heart transplant recipients.

## Introduction and background

Orthotopic heart transplantation (HTx) has been established as the gold standard for the management of advanced heart failure [1]. Major progress in pretransplant evaluation, perioperative management, immunosuppression, and long-term follow-up has markedly improved outcomes, with contemporary median survival now approaching 12.5 years in many adult cohorts [2]. Improving long-term survival has naturally expanded the goals of transplant care to include patients’ well-being in daily life. As a result, quality of life (QoL) has become one of the central considerations in evaluating the overall success of HTx, reflecting the understanding that survival alone does not fully capture the full spectrum of posttransplant health domains [3].

QoL is a multidimensional entity encompassing physical health, psychological well-being, social functioning, and treatment-related considerations [4]. Each of these dimensions may be significantly influenced by the transplant procedure itself and by the lifelong immunosuppressive therapy that follows. In recent years, psychological and emotional outcomes, particularly symptoms of anxiety and depression, have become integral components of comprehensive posttransplant evaluation because of their impact on adherence, coping, and long-term adjustment [5].

Numerous studies have demonstrated that QoL typically improves substantially after transplantation [6-8]. Although overall gains are well documented, the pace and extent of recovery vary across different domains of health. Physical capacity, emotional stability, social functioning, and treatment-related burden do not improve uniformly, and each may regain preillness or near-normal levels at different rates following surgery [3]. These variations are clinically meaningful, as trajectories in a specific QoL domain can influence long-term adjustment and may even contribute to differences in survival. Furthermore, QoL does not follow a single pattern for all individuals. Differences in follow-up duration, age at transplantation, and gender contribute to distinct recovery profiles, each with implications for long-term care and patient support.

This narrative review synthesizes current evidence on QoL in heart transplant recipients, with particular attention to validated measurement tools, physical, psychological, social, and treatment-related domains, and the evolution of QoL across early, intermediate, and long-term follow-up. By emphasizing temporal patterns and domain-specific outcomes, this review aims to provide a clinically oriented overview of how QoL changes after transplantation and which factors may influence long-term recovery.

## Review

Methods

Search Strategy

This narrative review was developed through a literature search of PubMed/MEDLINE and Scopus for studies evaluating QoL after HTx. Search terms included combinations of “heart transplantation,” “heart transplant recipients,” “quality of life,” “health-related quality of life,” “SF-36,” “WHOQOL-BREF,” “HADS,” “EQ-5D,” “psychological outcomes,” “social support,” “long-term survivors,” and “follow-up.”

Screening and Inclusion/Exclusion Criteria

Screening was performed by several authors based on title, abstract, and full-text relevance to the scope of the review. Studies were included if they evaluated adult heart transplant recipients and reported QoL, health-related QoL, psychological well-being, social functioning, treatment-related burden, functional recovery, or long-term posttransplant outcomes. Studies using validated QoL or psychological assessment tools, longitudinal studies, studies with larger sample sizes, randomized trials, meta-analyses, and studies reporting long-term follow-up were prioritized. Studies were excluded if they focused exclusively on non-heart transplant populations, did not report QoL or related functional/psychosocial outcomes, addressed only perioperative or survival outcomes without patient-reported or functional endpoints, or were not directly relevant to the domains discussed in this review.

Assessment of QoL: Instruments and Metrics

A meaningful evaluation of QoL after HTx requires the use of well-established and validated instruments; however, these tools differ in the domains they assess and may vary in their precision and relevance for specific aspects of well-being in heart transplant recipients [9,10]. Because QoL encompasses physical health, psychological state, social functioning, and treatment-related experiences, it is essential to rely on tools that assess each of these aspects in a structured and standardized way. These instruments provide quantifiable scores that allow clinicians and researchers to evaluate individual patients, compare findings across studies, and follow changes in QoL over time.

Short Form 36

The Short Form 36 (SF-36) is used to evaluate overall health status, capturing both physical and mental aspects of QoL. It consists of 36 items that generate quantitative scores across eight domains, which are combined into Physical and Mental Component scores. Although the instrument has demonstrated reliability and construct validity across cardiac and surgical populations, its psychometric performance and interpretation may vary depending on patient characteristics and the specific posttransplant context. Its applicability in transplant settings has been demonstrated in the work by Vojtěch Kurfirst and colleagues, who used the SF-36 to examine the association between preoperative health-related QoL and postoperative outcomes [11].

Giessen Subjective Complaints List-24

The Giessen Subjective Complaints List-24 (GBB 24) is used to assess subjective physical complaints that may influence overall QoL after HTx. It captures symptom clusters that commonly affect transplant recipients, including exhaustion, gastrointestinal discomfort, limb pain, and cardiac-related complaints. The questionnaire contains 24 items that generate quantitative subscale scores and a global discomfort score, with higher values indicating greater symptom burden [12,13]. The GBB-24 has demonstrated good internal consistency and reliability in chronic disease populations and was applied by Albert and colleagues in a cohort of 75 long-term HTx recipients 20-31 years after transplantation to compare subjective complaints with matched controls [6].

Social Support Questionnaire-Short Form With 14 Items

The Social Support Questionnaire-Short Form with 14 items measures perceived social support, an important determinant of psychological well-being and long-term adjustment after HTx. It includes 14 items assessing practical assistance, emotional support, and social integration. Higher scores reflect stronger perceived support. The tool has been validated in various medical and psychosocial contexts and is used in transplant research to characterize the role of social resources in shaping QoL trajectories [14].

EuroQol 5-Dimensions

The EuroQol 5-Dimensions (EQ-5D) provides a brief assessment of general health and is used when a concise global indicator of QoL is needed. It measures mobility, self-care, usual activities, pain or discomfort, and anxiety or depression, producing a single utility score and a visual analog scale rating [15,16]. In a longitudinal study of 38 patients with advanced heart failure evaluated before and at 3, 6, and 12 months after HTx, Almenar-Pertejo et al. used the EQ-5D to assess changes across these five dimensions and reported significant improvement after transplantation, with the most affected domains being usual activities and pain/discomfort [17].

World Health Organization Quality of Life-Short Form Questionnaire

The World Health Organization Quality of Life-Short Form Questionnaire assesses broader aspects of well-being, including physical health, psychological functioning, social relationships, and environmental factors. It provides quantitative domain scores on a 0-100 scale. The instrument is internationally validated and has been applied in selected HTx cohorts to characterize global QoL across physical, psychological, social, and environmental domains, particularly in studies examining long-term follow-up, treatment burden, socioeconomic factors, and differences between patient subgroups [18].

Hospital Anxiety and Depression Scale

The Hospital Anxiety and Depression Scale specifically evaluates emotional well-being by screening for symptoms of anxiety and depression through 14 nonsomatic items [19]. Its structure makes it appropriate for medically ill populations, including transplant recipients. It has strong reliability and validity across clinical settings and is frequently combined with general QoL instruments to assess the psychological component of post-HTx recovery [20,21].

Domains of QoL After HTx

Physical health domain: The physical health domain captures mobility, exercise tolerance, symptom burden, and the ability to perform daily activities. This domain often improves after transplantation as heart failure symptoms resolve, although the magnitude and pace of recovery may vary across studies, patient populations, and outcome measures. Exercise tolerance increases significantly after HTx, but peak oxygen uptake often remains well below age-matched normal levels because of persistent central and peripheral limitations, such as cardiac denervation and impaired skeletal muscle function [22]. In a randomized trial, Nytrøen et al. demonstrated that high-intensity interval training initiated early after transplant was safe and led to greater improvements in exercise capacity than moderate continuous training, alongside measurable gains in functional performance [23]. Consistent with this, European Society of Cardiology guidance describes cardiac rehabilitation after HTx as a phased process, including early in-hospital mobilization, structured rehabilitation after discharge, and long-term outpatient or home-based exercise programs to maintain functional recovery [24]. As for symptom burden, in the study by Stiefel et al., posttransplant symptoms were described without dyspnea being reported among them, suggesting that dyspnea may become less prominent after transplantation in selected cohorts [25]. However, fatigue and musculoskeletal discomfort persist in a substantial proportion of recipients and are frequently described as barriers to full physical recovery [4]. These residual symptoms, often related to long-term immunosuppression and deconditioning, highlight that physical recovery is substantial but not complete.

Psychological and Emotional Well-Being

The psychological domain encompasses emotional well-being, cognitive function, coping capacity, and behavioral responses that shape recovery after HTx. Although most recipients experience substantial relief once the burden of end-stage heart failure is removed, psychological distress remains common and does not uniformly resolve in parallel with physical recovery.

In a retrospective single-center cohort study of 114 adult heart transplant recipients, de la Rosa et al. reported posttransplant depression in 26.3% of patients during the first year after transplantation [26]. This underscores that emotional vulnerability may persist despite marked improvements in physical health. Pretransplant clinical pathways also influence posttransplant adjustment. Patients bridged to transplantation with mechanical circulatory support (MCS) have been reported to receive more intensive psychotherapeutic intervention than those transplanted without prior MCS, although this association may reflect greater pretransplant illness severity and psychosocial complexity rather than a direct effect of MCS itself [27]. Extended exposure to device-related stressors, prolonged hospitalization, and uncertainty during the bridging process likely contribute to this increased psychological burden.

Kinesiophobia, fear of movement due to anticipated pain or harm, further illustrates the interaction between psychological factors and physical recovery [28]. This fear often emerges in patients who experienced prolonged pre-transplant debilitation or distressing medical procedures and can persist after HTx, limiting engagement in rehabilitation even when graft function is stable. Addressing kinesiophobia is therefore important for supporting optimal functional recovery.

Cognitive function represents another component of psychological health with direct implications for daily living after HTx. Gaviria et al. recommend incorporating the Montreal Cognitive Assessment into routine evaluation, given that impairments in attention, memory, psychomotor processing, or executive functioning may compromise self-care and adherence [29]. In a study by Cho et al. evaluating cognitive outcomes after continuous-flow left ventricular assist device (LVAD) implantation, 668 patients completed baseline testing and at least one follow-up assessment. During follow-up, approximately 23% of tested patients showed a decline [30]. These findings suggest variable neurocognitive trajectories among patients supported with continuous-flow LVADs and may be relevant when evaluating early QoL after subsequent transplantation. Such cognitive challenges can influence emotional well-being, coping capacity, and the overall trajectory of psychological recovery.

Social Functioning and Reintegration

The social domain encompasses interpersonal relationships, participation in family and community life, and the ability to resume valued social or occupational roles. As physical symptoms resolve after transplantation, many recipients regain independence and reengage in social activities that were previously limited by advanced heart failure.

Return to employment, however, remains highly variable. While many recipients regain functional capacity, sustained workforce participation is limited. A large Danish registry study demonstrated that employment rates remain low several years after HTx, with vocational reintegration strongly influenced by multimorbidity and socioeconomic disadvantage [31]. These findings highlight that return-to-work outcomes depend not only on medical recovery but also on broader health and social determinants.

Social support remains one of the strongest predictors of favorable social and psychological outcomes. Support from spouses, family members, friends, and peers is consistently associated with higher emotional well-being and better global QoL. Long-term survivors who report strong perceived social support frequently demonstrate superior outcomes across multiple domains. A cross-sectional study by Gao et al. further showed that social support influences QoL through dual pathways: directly through strengthened relational networks and indirectly by reducing illness-related uncertainty through culturally shaped cognitive reframing [32]. These insights support the value of incorporating family-centered care and resilience-building strategies into long-term follow-up.

Treatment-Related Burden

The treatment-related domain reflects the cumulative impact of lifelong immunosuppression, medication side effects, and the monitoring required to prevent rejection and infection. Although HTx eliminates the hemodynamic burden of end-stage heart failure, it introduces a demanding therapeutic regimen that can meaningfully influence long-term QoL.

Immunosuppressive therapy remains the major contributor to treatment-related burden [33,34]. Calcineurin inhibitors, corticosteroids, and adjunctive agents are essential for graft protection but are associated with renal dysfunction, hypertension, metabolic disturbances, neuropsychiatric symptoms, cosmetic effects, and increased infection risk. Polypharmacy, frequent monitoring, and the long-term vigilance required for medication adherence add practical and emotional strain for many recipients.

Adjustments in immunosuppression can modify both objective outcomes and patient experience. In a randomized clinical trial, Faulhaber et al. [35] demonstrated that steroid withdrawal combined with reduced cyclosporine exposure and mycophenolate mofetil improved renal and cardiovascular parameters in stable long-term recipients, yet some patients experienced subjective deterioration and withdrawal-related symptoms, illustrating that regimen optimization may carry both benefits and burdens.

Broader strategies to refine immunosuppression also influence this QoL domain. Eisen and Ross emphasized that tailoring immunosuppressive therapy to reduce cardiovascular risk and mitigate long-term toxicities is central to preventing cardiac allograft vasculopathy [36]. Approaches include minimizing cumulative corticosteroid and calcineurin inhibitor exposure, incorporating newer agents such as everolimus or sirolimus with demonstrated efficacy against vasculopathy, and improving drug monitoring techniques to reduce side effects while maintaining adequate immunosuppression. These evolving strategies underscore that treatment-related burden is shaped not only by medication effects but also by therapeutic choices aimed at protecting long-term graft health.

Evolution of QoL Over Time: Early, Intermediate, and Long Term

QoL after HTx follows a dynamic and phase-dependent trajectory. Distinct patterns emerge in the early posttransplant period, the intermediate term (one to five years), and the long-term period beyond 5-10 years. Although improvements are substantial, trajectories vary widely and are influenced by factors such as age, comorbidities, psychosocial resources, pretransplant clinical course, and posttransplant complications.

Early Posttransplant Period (First Year)

The first year after HTx is marked by rapid and substantial improvement in QoL, with the largest gains seen within the first three to six months. As heart failure symptoms resolve, physical functioning, vitality, and general health rise quickly, and by three months, no domain remains as impaired as before transplant. By 6-12 months, most recipients are independent in daily activities and have adapted to their medication regimen, establishing a new and significantly improved baseline of health [37].

Alongside these physical improvements, patients undergo an emotionally demanding adjustment period [38]. Many experience fear of complications and rejection, ongoing uncertainty about recovery, and episodes of anxiety that may strain interpersonal interactions. At the same time, recipients frequently describe profound gratitude and renewed hope, often viewing the transplant as a second chance at life. Adapting to strict treatment routines, managing side effects, and reassessing early expectations for progress can create tension between anticipated and actual recovery. Some individuals also report temporary social withdrawal as they cautiously resume work, social roles, or daily activities. Importantly, these emotional and cognitive responses are not peripheral but integral to early outcomes. Grady et al. [39] demonstrated that predictors of one-year QoL were predominantly psychological, underscoring that emotional resilience, coping strategies, and perceived support play a central role in shaping overall well-being in the first year after transplant.

Intermediate Term (One to Five Years Posttransplant)

Between one and five years after HTx, QoL generally remains high and stable among recipients with well-functioning grafts. The substantial gains achieved during the first postoperative year are largely maintained, and many individuals report sustained recovery in physical capacity, emotional well-being, and day-to-day functioning. Several longitudinal studies show that self-reported health and global QoL remain essentially unchanged between one, three, and five years, reflecting the durability of early improvements [40]. Physical functioning typically remains robust in this period. As confidence grows and rehabilitation continues, many patients increase their activity levels, with some returning to exercise or employment during the second and third years. Mental health also tends to stabilize; initial fears surrounding rejection and early complications diminish with time as patients adapt to living with a transplanted heart. However, some differences persist when comparing transplant recipients to population norms [41]. In several analyses, both physical and mental health scores remain slightly lower than those of age-matched healthy controls, suggesting residual effects of chronic illness, immunosuppression, or ongoing medical surveillance. Additionally, the intermediate period marks the emergence of longer term complications such as cardiac allograft vasculopathy, chronic kidney disease, diabetes, malignancy, and persistent medication side effects [42]. These issues may lead to small declines in QoL for affected individuals, although the overall group trajectory remains stable.

Long-Term Period (Beyond One Decade After Transplantation)

Long-term QoL outcomes beyond 10 years after HTx are less extensively documented, and the available evidence is limited and potentially affected by survivor bias, as findings largely reflect selected cohorts who have survived into the second and third posttransplant decades.

A study by Albert et al. [6] evaluating recipients 20-31 years after transplantation showed that most maintained preserved ventricular function and remained in functional class I or II. Compared with matched controls, these long-term survivors reported lower scores in the physical component of QoL and more subjective physical complaints, while mental health scores and emotional stability were similar to control levels. Cluster analysis identified distinct subgroups differentiated by perceived social support, underscoring the continuing relevance of interpersonal resources many years after transplantation.

Findings from Galeone et al. [43] further support this pattern. In their cohort, approximately 16% of transplant recipients survived for 20 years or longer, with preserved ventricular performance and stable perceived QoL. Despite substantial late morbidity, including cardiac allograft vasculopathy and malignancies, psychological well-being remained relatively intact. Additional insight comes from a descriptive report by Javier Delmo et al. [44] of recipients more than 30 years after transplantation. These individuals described active and satisfying daily lives with a sense of meaning and social connectedness. Although chronic conditions related to immunosuppression were common, the reported emotional state remained positive.

Overall, these studies suggest that physical limitations and chronic complications, including cardiac allograft vasculopathy, renal dysfunction, malignancies, and metabolic disturbances, may become more prominent over extended follow-up. In contrast, mental and social well-being may remain relatively stable in selected long-term survivors. While these findings should be interpreted in the context of survivor selection, the available evidence indicates that patients who reach extended posttransplant milestones can maintain durable QoL despite accumulating health challenges.

## Conclusions

As efforts to refine posttransplant survival outcomes continue, QoL has become an increasingly important focus in HTx research. A growing body of evidence demonstrates meaningful associations between QoL and key clinical outcomes, underscoring that long-term success after transplantation depends not only on graft longevity but also on patients’ functional, psychological, and social well-being. Despite this progress, robust long-term QoL data remain limited, with relatively few studies extending beyond the first decade after transplantation. Expanding high-quality, longitudinal research will be essential to better characterize late trajectories, identify modifiable determinants of QoL, and inform future strategies aimed at optimizing both survival and lived experience for heart transplant recipients.

## References

[1] Writing Committee Members; ACC/AHA Joint Committee Members (2022). 2022 AHA/ACC/HFSA guideline for the management of heart failure. J Card Fail.

[2] Aguilar J, Miller RJ, Otaki Y (2022). Clinical utility of SPECT in the heart transplant population: analysis from a single large-volume center. Transplantation.

[3] Emin A, Rogers CA, Banner NR (2016). Quality of life of advanced chronic heart failure: medical care, mechanical circulatory support and transplantation. Eur J Cardiothorac Surg.

[4] Mahmoudi R, Battistella P, Sebbag L, Adoli LK, Guillemin F, Couchoud C (2025). Perceptions of health-related quality of life among heart transplant recipients: a qualitative study. Transplant Proc.

[5] Bürker BS, Malt UF, Gude E, Grov I, Relbo Authen A, Dew MA, Gullestad L (2021). Symptoms of anxiety after heart transplantation and their association with mortality: a secondary analysis. Clin Transplant.

[6] Albert W, Hudalla A, Hensky L, Akın A, Knosalla C, Richter F (2024). Quality of life in patients 20-31 years after heart transplantation. Clin Transplant.

[7] Mahmoudi R, Moitie T, Dorent R, Guillemin F, Couchoud C (2022). Implementation of patient-reported outcome measures in a heart transplant recipient registry: first step toward a patient-centered approach. Clin Transplant.

[8] Rimmer B, Jenkins R, Russell S, Craig D, Sharp L, Exley C (2024). Assessing quality of life in solid organ transplant recipients: a systematic review of the development, content, and quality of available condition- and transplant-specific patient-reported outcome measures. Transplant Rev (Orlando).

[9] Laviña SM (2024). Measuring quality of life through validated tools. Acta Med Philipp.

[10] Mahmoudi R, Camille L, Guillemin F, Couchoud C (2026). Feasibility and acceptance of electronic quality of life assessment in heart transplant recipients. Transplant Rep.

[11] Kurfirst V, Mokráček A, Krupauerová M, Canádyová J, Bulava A, Pešl L, Adámková V (2014). Health-related quality of life after cardiac surgery--the effects of age, preoperative conditions and postoperative complications. J Cardiothorac Surg.

[12] Brähler E, Schumacher J, Brähler C (2000). First nationwide standardization of the short form of the Giessen Complaint Form GBB-24. Psychother Psychosom Med Psychol.

[13] Brähler E, Schumacher J, Brähler C (1999). First Pan-German standardization and specific validity aspects of the Giessen test. Z Differ Diagn Psychol.

[14] Reinwarth AC, Petersen J, Beutel ME, Hautzinger M, Brähler E (2024). Social support in older adults: validation and norm values of a brief form of the Perceived Social Support Questionnaire (F-SozU K-6). PLoS One.

[15] Devlin N, Parkin D, Janssen MF (2020). An introduction to EQ-5D instruments and their applications. Methods for Analysing and Reporting EQ-5D Data.

[16] McDonald R, Mullett TL, Tsuchiya A (2020). Understanding the composite dimensions of the EQ-5D: an experimental approach. Soc Sci Med.

[17] Almenar-Pertejo M, Almenar L, Martínez-Dolz L (2006). Study on health-related quality of life in patients with advanced heart failure before and after transplantation. Transplant Proc.

[18] Carvalho WD, Alves Maria GD, Gonçalves KC, Miranda AL, Moreira MD (2021). Health-related quality of life of heart transplant recipients living in a developing country. Transplant Proc.

[19] Mitchell AJ, Meader N, Symonds P (2010). Diagnostic validity of the Hospital Anxiety and Depression Scale (HADS) in cancer and palliative settings: a meta-analysis. J Affect Disord.

[20] Bunzel B, Laederach-Hofmann K, Wieselthaler GM, Roethy W, Drees G (2005). Posttraumatic stress disorder after implantation of a mechanical assist device followed by heart transplantation: evaluation of patients and partners. Transplant Proc.

[21] Sánchez R, Baillès E, Bastidas A, Pérez-Villa F, Bulbena A, Pintor L (2015). Prospective psychiatric assessment of a cohort of Spanish heart transplant recipients. Rev Esp Cardiol (Engl Ed).

[22] Tucker WJ, Beaudry RI, Samuel TJ, Nelson MD, Halle M, Baggish AL, Haykowsky MJ (2018). Performance limitations in heart transplant recipients. Exerc Sport Sci Rev.

[23] Nytrøen K, Rolid K, Andreassen AK (2019). Effect of high-intensity interval training in de novo heart transplant recipients in Scandinavia. Circulation.

[24] Piepoli MF, Corrà U, Adamopoulos S (2014). Secondary prevention in the clinical management of patients with cardiovascular diseases. Core components, standards and outcome measures for referral and delivery: a policy statement from the cardiac rehabilitation section of the European Association for Cardiovascular Prevention & Rehabilitation. Endorsed by the Committee for Practice Guidelines of the European Society of Cardiology. Eur J Prev Cardiol.

[25] Stiefel P, Malehsa D, Bara C, Strueber M, Haverich A, Kugler C (2013). Symptom experiences in patients after heart transplantation. J Health Psychol.

[26] de la Rosa A, Singer-Englar T, Hamilton MA, IsHak WW, Kobashigawa JA, Kittleson MM (2021). The impact of depression on heart transplant outcomes: a retrospective single-center cohort study. Clin Transplant.

[27] Heilmann C, Kuijpers N, Beyersdorf F (2012). Does listing for heart transplant for longer than 30 days before ventricular assist device implantation influence utilization of psychotherapeutic support and outcome?. Eur J Cardiothorac Surg.

[28] Marques-Sule E, Cabanillas-García JL, Almenar-Bonet L (2025). Kinesiophobia, physical limitations and psychological distress as barriers to physical activity in heart transplantation patients: a qualitative study. J Clin Med.

[29] Gaviria M, Pliskin N, Kney A (2011). Cognitive impairment in patients with advanced heart failure and its implications on decision-making capacity. Congest Heart Fail.

[30] Cho SM, Floden D, Wallace K (2021). Long-term neurocognitive outcome in patients with continuous flow left ventricular assist device. JACC Heart Fail.

[31] Mols RE, Løgstrup BB, Bakos I, Horváth-Puhó E, Gustafsson F, Eiskjær H (2024). Employment status following heart transplantation: data from the Danish nationwide social service payment register during 20 years. Transpl Int.

[32] Gao C, Gui S, Zhu L, Bian X, Shen H, Jiao C (2025). Social support and quality of life in Chinese heart transplant recipients: mediation through uncertainty in illness and moderation by psychological resilience. Front Psychiatry.

[33] Habal M (2025). Immunosuppression management in heart transplantation. Methodist Debakey Cardiovasc J.

[34] Chang DH, Kittleson MM, Kobashigawa JA (2014). Immunosuppression following heart transplantation: prospects and challenges. Immunotherapy.

[35] Faulhaber M, Mäding I, Malehsa D, Raggi MC, Haverich A, Bara CL (2013). Steroid withdrawal and reduction of cyclosporine A under mycophenolate mofetil after heart transplantation. Int Immunopharmacol.

[36] Eisen H, Ross H (2004). Optimizing the immunosuppressive regimen in heart transplantation. J Heart Lung Transplant.

[37] Myaskovsky L, Dew MA, McNulty ML, Switzer GE, DiMartini AF, Kormos RL, McCurry KR (2006). Trajectories of change in quality of life in 12-month survivors of lung or heart transplant. Am J Transplant.

[38] Nour El Hadi S, Zanotti R, Danielis M (2025). Lived experiences of persons with heart transplantation: a systematic literature review and meta-synthesis. Heart Lung.

[39] Grady KL, Jalowiec A, White-Williams C (1999). Predictors of quality of life in patients at one year after heart transplantation. J Heart Lung Transplant.

[40] Politi P, Piccinelli M, Fusar-Poli P (2004). Ten years of "extended" life: quality of life among heart transplantation survivors. Transplantation.

[41] Rosenberger EM, Fox KR, DiMartini AF, Dew MA (2012). Psychosocial factors and quality-of-life after heart transplantation and mechanical circulatory support. Curr Opin Organ Transplant.

[42] Coutance G, Kobashigawa JA, Kransdorf E, Loupy A, Desiré E, Kittleson M, Patel JK (2023). Intermediate-term outcomes of complement inhibition for prevention of antibody-mediated rejection in immunologically high-risk heart allograft recipients. J Heart Lung Transplant.

[43] Galeone A, Kirsch M, Barreda E (2014). Clinical outcome and quality of life of patients surviving 20 years or longer after heart transplantation. Transpl Int.

[44] Javier Delmo EM, Javier MF, Wagner F, Hetzer R (2021). Resumé of the challenges and burden of living with transplanted hearts for >31 years. Cardiovasc Diagn Ther.

